# Polygenic risk scores indicates genetic overlap between peripheral pain syndromes and chronic postsurgical pain

**DOI:** 10.1007/s10048-020-00614-5

**Published:** 2020-05-06

**Authors:** Roel R. I. van Reij, Jan Willem Voncken, Elbert A. J. Joosten, Nynke J. van den Hoogen

**Affiliations:** 1 Department of Anaesthesiology and Pain Management, Maastricht University Medical Center+, 6200 MD Maastricht, The Netherlands; 2 Department of Translational Neuroscience, School for Mental Health and Neuroscience (MHeNs), University of Maastricht, 6200 MD Maastricht, The Netherlands; 3 Department of Molecular Genetics, Maastricht University Medical Center+, 6200 MD Maastricht, The Netherlands

**Keywords:** CPSP, GWAS, PRS, SNPs, Gene ontology

## Abstract

Chronic postsurgical pain (CPSP) is a debilitating chronic pain condition that has a substantial effect on quality of life. CPSP shows considerable clinical overlap with different chronic peripheral pain syndromes, suggesting a shared aetiology. This study aims to assess the genetic overlap between different chronic pain syndromes and CPSP, providing relevant biological context for potential chronic pain markers of CPSP. To analyse the genetic overlap between CPSP and chronic peripheral pain syndromes, recent GWAS studies were combined for polygenic risk scores (PRS) analysis, using a cohort of CPSP patients as starting point. Biological contextualisation of genetic marker, overlap between CPSP and chronic pain syndromes, was assessed through Gene Ontology (GO), using Pathway Scoring Algorithm (PASCAL) and REVIGO. PRS analyses suggest a significant genetic overlap between CPSP and 3 chronic pain disorders: chronic widespread pain (CWP, *p* value threshold = 0.003, *R*^2^ 0.06, *p* = 0.003), rheumatoid arthritis (RA, *p* value threshold = 0.0177, *R*^2^ = 0.04, *p* = 0.017) and possibly sciatica (*p* value threshold = 0.00025, *R*^2^ = 0.03, *p* = 0.045). Whereas no significant genetic overlap was found with cluster headache and migraine, the outcome for osteoarthritis (OA) was inconsistent between the cohorts. This is likely related to cohort composition, as repeated random reallocation of patients’ nullified CPSP/OA outcome variation between the discovery and replication cohorts. GO analyses suggested an aetiological involvement of genetic markers that control neurological signalling (specifically sodium channels) and inflammatory response. The current study reaffirms the impact of sample size, cohort composition and open data accessibility on the unbiased identification of genetic overlap across disorders. In conclusion, this study is the first to report genetic overlap between regulatory processes implicated in CPSP and chronic peripheral pain syndromes. Interaction between neurological signalling and inflammatory response may explain the genetic overlap between CPSP, CWP and RA. Enhanced understanding of mechanisms underlying chronification of pain will aid the development of new therapeutic strategies for CPSP with sodium channel biochemistry as a potential candidate.

**Electronic supplementary material:**

The online version of this article (10.1007/s10048-020-00614-5) contains supplementary material, which is available to authorized users.

## Introduction

Chronic postsurgical pain (CPSP) is a debilitating chronic pain condition that affects patients who underwent surgery and has a substantial effect on the quality of life (QoL) and socioeconomic status [1–3]. CPSP is defined as “*pain developed or increased after a surgical procedure, which is present for at least three months, and affecting the QoL*” [4, 5]. Depending on the type of surgery, 5–85% of the patients may experience pain localized to the surgical field or the projected innervation area of a nerve [4, 6]. Clinical (e.g. type/duration of surgery), demographical (e.g. age, biological sex) and psychological (e.g. anxiety) risk factors of CPSP can account for 78% of the variance in the development of CPSP [7, 8]. Although recent evidence (both GWAS and gene-targeted studies) defines a potential role for genetic risk factors in CPSP, the limited CPSP sample size in comparison with studies of other chronic pain syndromes has thus far not provided clear candidate genes for CPSP [9–12]. While increasing sample size for GWAS analyses holds the potential for unambiguous identification of genetic risk factors, genetic mechanisms underlying CPSP may be probed indirectly by determination of common genetic factors with other pain syndromes. Polygenic risk scores (PRS) allow testing for genetic correlation (i.e. overlap) between different phenotypes [13, 14]. Establishing the intersection and/or overlap of genetic networks between various chronic pain syndromes may help define common mechanisms in chronic pain and provide starting points for functional and intervention studies.

CPSP shows considerable overlap with different chronic peripheral pain syndromes (CPPs), among which sciatic pain, chronic widespread pain, osteoarthritis and rheumatoid arthritis with regard to demographical and psychological risk factors: chronic pain occurs most often in women, is associated with age and with psychological syndromes [15–17].

Although such observational studies suggest a shared aetiology between CPSP and chronic peripheral pain syndromes, the identity and interplay of underlying genetic causes and molecular processes that contribute to chronic pain, are incompletely understood [15–17]. Therefore, this study aims to assess whether different chronic pain syndromes show genetic overlap with CPSP and to provide relevant biological context for potential genetic risk factors. Ultimately, identification of novel targets is expected to pave the way for a better understanding of cellular and molecular mechanisms in CPSP and provide therapeutic opportunities.

## Methods

To assess whether chronic pain syndrome show genetic overlap with CPSP, we assessed several available datasets against a discovery and replication cohort of CPSP patients. The protocol for this study was reviewed and approved by the local Medical Ethical Committees (both discovery and replication study); all participants have provided written informed consent. The discovery cohort was registered at the Dutch trial registry under the number NTR2702 (http://www.trialregister.nl/trialreg/index.asp). The replication cohort was registered at the Clinical Trials registry under the number NCT02002663 and NCT01989351 (https://clinicaltrials.gov/ct2/home).

### Genome-wide association analysis

An elaborate description of patient recruitment, sample and data collection protocols for the discovery and replication cohorts has been published elsewhere [8, 9, 18]. In brief, a multicentre cohort study was conducted in four hospitals in the Netherlands (discovery cohort, *n* = 303) and three hospitals in Italy (replication cohort, *n* = 77). DNA-samples were genotyped at the Department of Genomics at the Life and Brain Center, University of Bonn using the Illumina PsychArray (Infinium PsychArray-24 v1.2 Bead Chip, Illumina Inc., USA). Genotypes were called using BeadStudio (Genome Studio v2011.1, Illumina). Basic quality control was done using Plink (Plink-1.9) [19, 20]. The quality control parameters consisted of: SNP call rate < 0.95, subject call rate of < 0.95, deviation of Hardy-Weinberg equilibrium (*P* < 1 × 10^−6^) and removal of rare variants with a minor allele frequency < 0.01. Heterozygosity of the subjects was tested, and outliers (± 3 SD from the mean heterozygosity rate) were removed. Genotype imputation was performed using the stepwise imputation approach implemented in Minimac3 (https://genome.sph.umich.edu/wiki/Minimac3; University of Michigan, Ann Arbor, USA) and Eagle2 (https://data.broadinstitute.org/alkesgroup/Eagle/; Broad Institute, Cambridge, USA v2.3) using default parameter settings and a European HRC reference panel (http://www.haplotype-reference-consortium.org/; version r1.1 2016) [21–23].

GWAS was carried out using SNPTEST (https://mathgen.stats.ox.ac.uk/genetics_software/snptest/snptest.html; Oxford University, Oxford, United Kingdom, v2.5.4) [24, 25]. The primary outcome measured in the discovery cohort was the highest surgery-related pain score measured by a numeric rating scale (NRS), recorded at rest during the last week, 3 months postsurgery [8, 9]. Based on the primary outcome measure, patients were divided into a nonCPSP (NRS = 0) and a CPSP (NRS > 3) group.

### Cohort selection for polygenic risk score calculation

To analyse the genetic overlap between CPSP and chronic peripheral pain syndromes, recent GWAS studies were used to form PRS scores in order to differentiate between patients who developed CPSP and those who did not. Using PubMed, we identified 6 GWAS reports on chronic pain syndromes (sciatic pain, migraine, chronic widespread pain, osteoarthritis, rheumatoid arthritis, and cluster headache) meeting the inclusion criteria [11, 12, 26–30]: The headache-related disorders (migraine and cluster headache) were selected as negative control due to a different pathophysiology [31, 32]. A total of 7208 SNPs were reported as summary statistics in migraine, of which 214 were present after pruning in the discovery cohort and 207 after pruning in the replication cohort [11]. A total of 14,167 SNPs were reported as summary statistics in cluster headache, of which 6906 were present after pruning in the discovery cohort and 7438 after pruning in the replication cohort [28]. Eighty-nine SNPs were reported as summary statistics in chronic widespread pain, of which 34 were present after pruning in the discovery cohort and 35 after pruning in the replication cohort [26]. One hundred twenty-nine SNPs were reported as summary statistics in osteoarthritis, of which 74 were present after pruning in the discovery cohort and 76 after pruning in the replication cohort [27]. A total of 297,081 SNPs were reported as summary statistics in rheumatoid arthritis, of which 50,294 were present after pruning in the discovery cohort and 51,834 after pruning in the replication cohort [29]. A total of 380,066 SNPs were reported as summary statistics in sciatica, of which 20,744 were present after pruning in the discovery cohort and 19,053 after pruning in the replication cohort [12]. All SNPs included in the analysis per study per cohort can be found in Supplementary file 1.

### Shared genetic background analysis

The polygenic risk score analysis tool PRSice was used to determine genetic overlap between chronic pain syndromes and CPSP [33]. Summary statistics of published studies on chronic pain syndromes were used as ‘reference dataset’ and the data of the discovery and replication cohorts after quality control (described in the original publication) as ‘target phenotype’ sample [9, 11, 12, 26–29]. The target phenotype was considered a dichotomous variable, defined as presence of CPSP (yes or no), and the base phenotypes were used to differentiate between presence and absence of CPSP.

PRS analysis settings comprised pruning based on linkage disequilibrium (*r*^2^ > 0.1) within a 250 kb window and incrementally increasing summary statistic *p* value threshold starting at *p* < 0.0001 (increasing with increments of 0.00005) [34]. This determines optimal SNPs fit with regard to predicting polygenic risk score. Identical parameters were used for the discovery and replication cohorts.

### Pathway analysis

Biological context for potential genetic overlap between CPSP and chronic pain syndromes was assessed using pathway scoring algorithm (PASCAL) [35]. The input data consisted of all SNPs of significant PRSs for both the discovery cohort and the replication cohort using *p* values reported in the original publications [12, 26, 29]. Pathway scoring was done using the biological processes (BPGO), molecular function (MFGO) and cell component (CCGO) databases of the gene ontology resource (GO) [36, 37]. Pathway enrichment was assessed by comparing enrichment score of the provided gene sets with a random sampling permutation–based distribution per pathway. To correct for multiple testing, the empirical *p* values of the PASCAL enrichment were corrected using the p.adjust function with false discovery rate (FDR) in *R* [38, 39]. Clustering of GO terms were visualized using REVIGO based on GO id’s and PASCAL *p* values with similarity set to small, similarity measure to SimRel and using the uniport database as a reference [40].

### Statistics

GWAS data was analysed using logistic regression and the *p* values were corrected for the number of SNPs analysed using Bonferroni correction. PRSice was used to determine polygenic risk scores of SNPs obtained from analysis of the base dataset weighted by their respective effect sizes [34]. The PRS scores were calculated assuming an additive model with the following equation:

$$ \mathrm{PRS}j=\frac{\mathrm{S}i\times \mathrm{G} ij}{\mathrm{M}j} $$

where *S* denotes the summary statistics for the effective allele of SNP *i*, G denotes the number of effective alleles observed for individual *j* for SNP *i* and M denotes the number of alleles included in the PRS of the individual *j.* Significance was set at *p* ≤ 0.05.

All graphs were visualized using *R* [39].

## Results

### Analysis of genetic overlap between chronic pain syndromes and CPSP discovery cohort

**Fig. 1:**
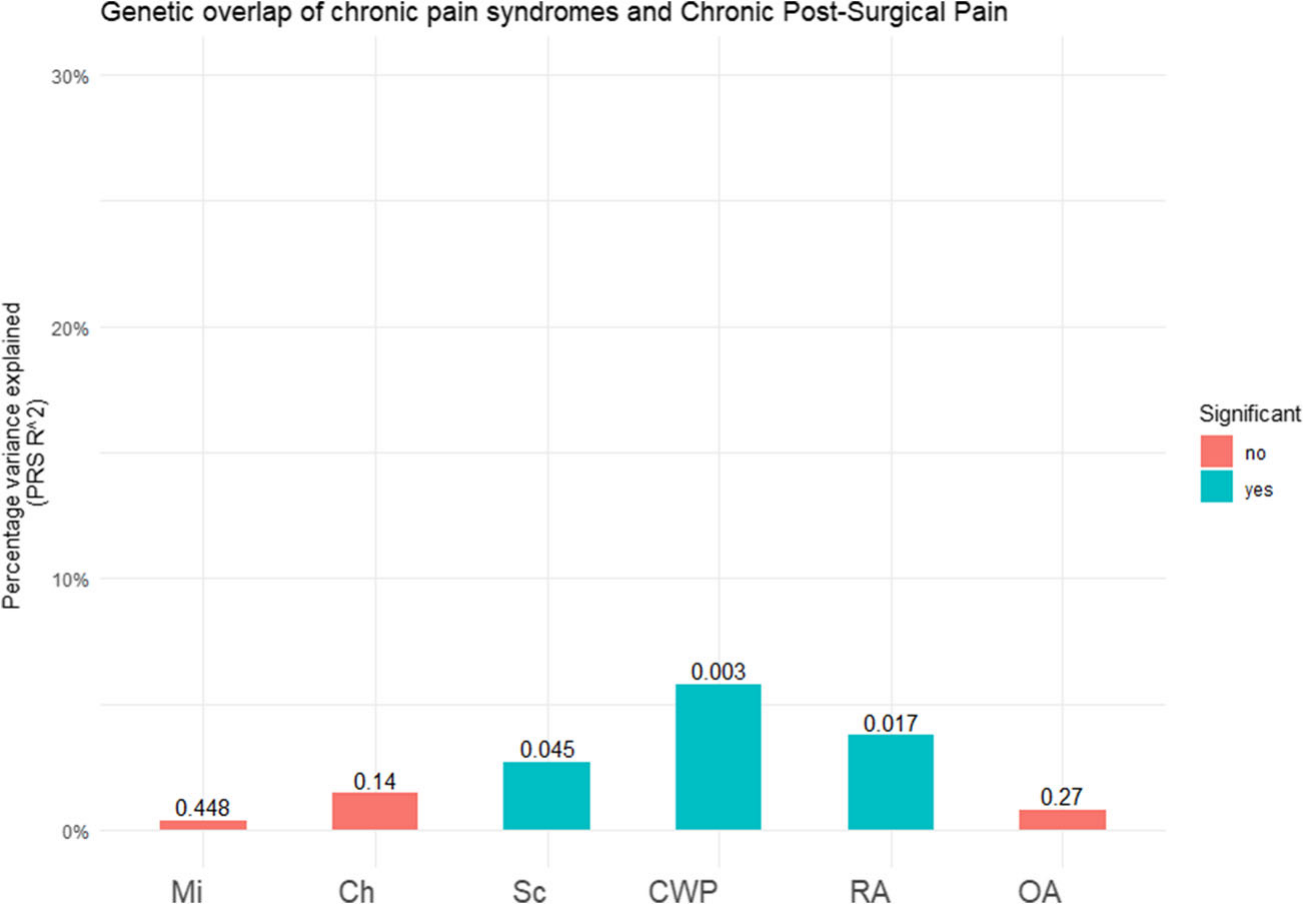
Genetic overlap of chronic pain syndromes and chronic postsurgical pain in discovery cohort. Graphic representation of the genetic overlap between different chronic pain syndromes and CPSP discovery cohort. *Y*-axis depicts variance explained by the polygenic risk score, *x*-axis depicts the different phenotypes and the numbers indicate the *p* values of the polygenic risk scores. Mi = migraine, Sc = sciatica, CWP = chronic widespread pain, RA = rheumatoid arthritis, OA = osteoarthritis

PRS was used to assess genetic overlap between the chronic pain phenotypes and the discovery cohort of CPSP. A significant genetic overlap was found between 3 chronic pain disorders and CPSP: chronic widespread pain (*p* value threshold = 0.003, *R*^2^ 0.06, *p* = 0.003) and rheumatoid arthritis (*p* value threshold = 0.0177, *R*^2^ = 0.04, *p* = 0.017) and Sciatica (*p* value threshold = 0.00025, *R*^2^ = 0.03, *p* = 0.045). No significant genetic overlap was found with osteoarthritis, cluster headache and migraine (Fig. 1, Supplementary Table 1). This finding suggested significant genetic overlap between sciatica, chronic widespread pain and rheumatoid arthritis and CPSP but no genetic overlap between cluster headache, migraine and osteoarthritis and CPSP.

### Validation of genetic overlap in CPSP replication cohort

**Fig. 2:**
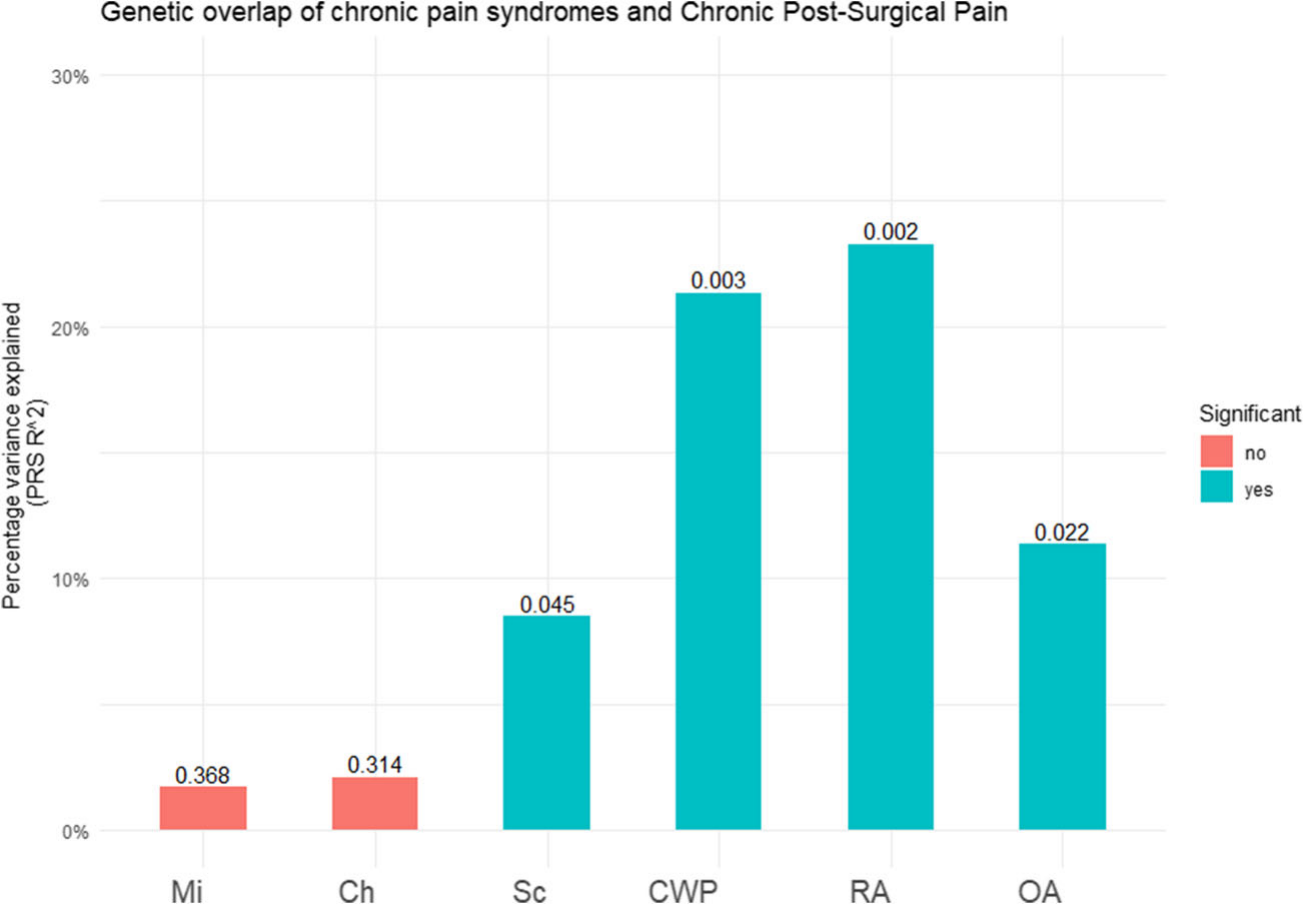
Genetic overlap of chronic pain syndromes and chronic postsurgical pain in replication cohort. Graphic representation of the genetic overlap between three chronic pain syndromes and CPSP replication cohort. *Y*-axis depicts variance explained by the polygenic risk score, *x*-axis depicts the different phenotypes and the numbers indicate the *p* values of the polygenic risk scores. Mi = migraine, Sc = sciatica, CWS = chronic widespread pain, RA = rheumatoid arthritis, OA = osteoarthritis

To validate the discovery cohort based findings on genetic overlap between CPSP and chronic pain syndromes, the PRS analysis was independently repeated in the replication cohort. Although, the percentage variance explained by PRS was a factor 3–4 higher in the replication cohort, consistent with the outcome of the discovery cohort, a significant genetic overlap was observed with three of the chronic pain disorders and CPSP: Sciatica (*p* value threshold = 0.00385, *R*^2^ = 0.08, *p* = 0.045), chronic widespread pain (*p* value threshold = 0.141, *R*^2^ 0.21, *p* = 0.0003), rheumatoid arthritis (*p* value threshold = 0.3549, *R*^2^ = 0.23, *p* = 0.002; Fig. 2, Supplementary Table 2); In addition, PRS analysis of the replication cohort produced significant overlap with osteoarthritis (*p* value threshold = 0.0001, *R*^2^ = 0.11, *p* = 0.022) (Fig. 2, Supplementary Table 2). No significant genetic overlap was found between cluster headache and migraine and CPSP.

### Pathway analysis of genes associated with significant polygenic risk scores

**Fig. 3:**
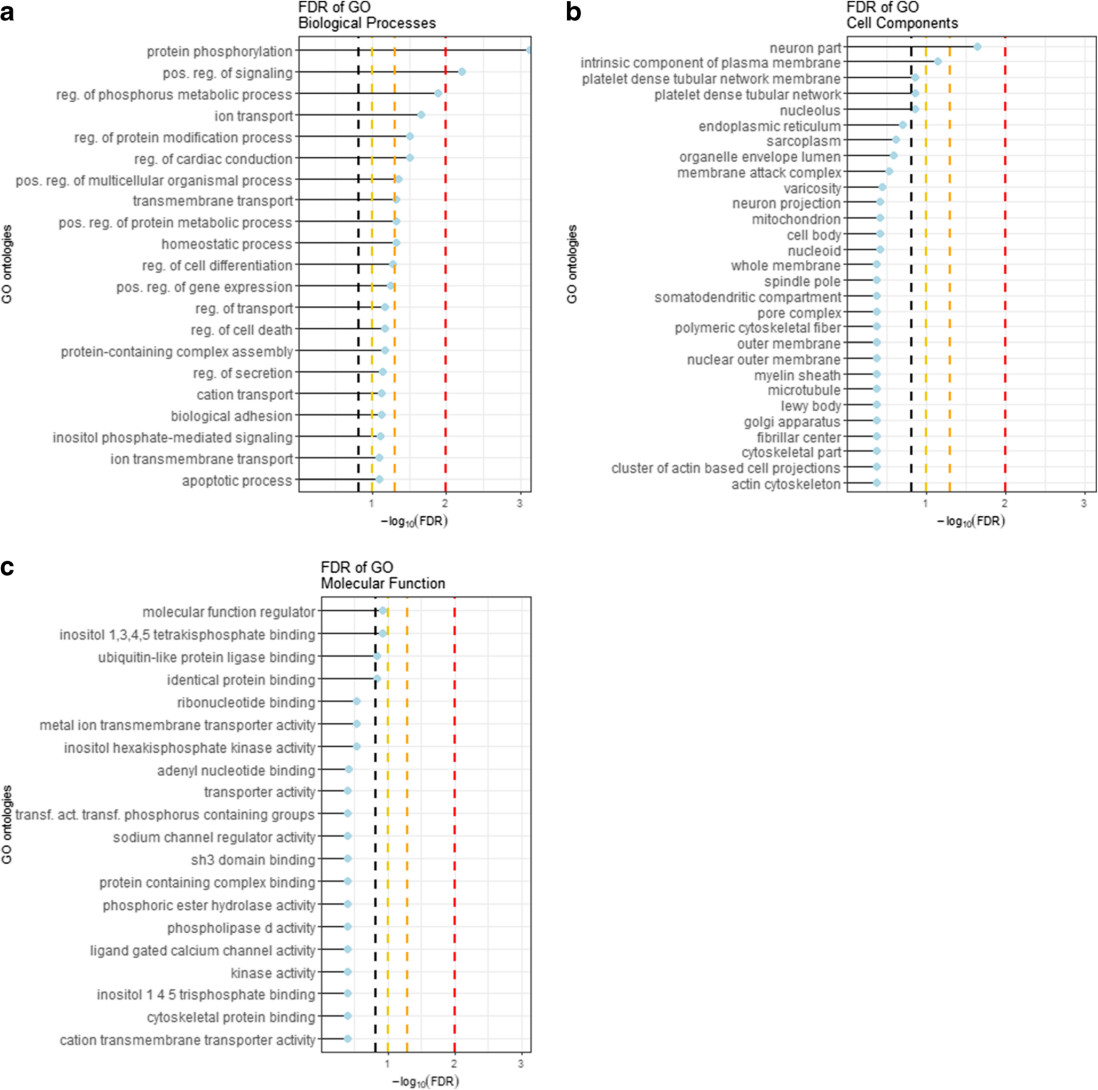
Graphic representation of GO analyses on genetic factors with significant PRS scores. Lollipop plots represent the top 20 associations of GO terms with the respective GO databases. Dotted lines represent FDRs of 15% (black), 10% (yellow), 5% (orange) and 1% (red), respectively

To assess the biological context defined by common genetic markers of chronic pain disorders and CPSP, an exploratory pathway analysis was performed cohorts using pathway scoring algorithm (PASCAL) [35]. In agreement with published instruction, pathway analysis was limited to SNPs that showed significant genetic overlap in both the discovery and replication cohorts [35]. The findings of the PASCAL analysis were clustered using REVIGO [40]. BPGO revealed enrichment for 3 terms at FDR < 1%, 17 terms at FDR ≤ 5%, 9 terms at FDR ≤ 10% and 26 terms at FDR ≤ 15% (Fig. 3a, Supplemental Table 3). These terms clustered into 4 main clusters: protein phosphorylation, positive regulation of signalling, response to cytokine and cation transport (Supplemental Fig. 1). CCGO revealed enrichment for 1 term at FDR < 1%, and 1 term at FDR < 5% (Fig. 3b, Supplemental Table 4). The terms associated with cellular components clustered into 3 main clusters: endoplasmic reticulum, sodium channel complex and intrinsic component of plasma membrane (Supplemental Fig. 2). MFGO revealed enrichment for 3 terms at FDR < 10% (Fig. 3c, Supplementary Table 5). The terms associated with molecular functions clustered into 5 main clusters: identical protein binding, phosphoric ester hydrolase activity, metal ion transmembrane transporter activity, phosphatidylinositol binding and sodium channel regulator activity (Supplemental Fig. 3). Taken together, the clusters identified by the GO analyses suggested an aetiological involvement of genetic markers that control neurological signalling and inflammatory response.

## Discussion

The present report is the first to study genetic overlap between different chronic peripheral pain syndromes (CPPs) and CPSP based on polygenic risk score (PRS) analysis. We hypothesised that CPSP shares biological mechanisms and hence genetic factors with some of the known chronic pain syndromes, among which chronic widespread pain and rheumatoid arthritis. Polygenic risk score analyses showed significant genetic overlap between CPSP and CPPs (chronic widespread pain, rheumatoid arthritis and possibly with sciatica, but not with osteoarthritis or common headache phenotypes (migraine or cluster headache). Functional enrichment analysis using PASCAL and REVIGO implicate the genes identified in the genetic overlap to be involved in regulation of neurological signalling and inflammatory response.

The scale of variance explained by genetic overlap differed between the discovery and replication cohort. This likely relates to the difference in sample size, as small sample sizes tend to inflate the explained variance. The size of the target sample (the sample size of the discovery cohort was roughly three to four times the size of the replication cohort) is correlated with the reliability of the variance explained [41]. In addition, variation in numbers of reported SNPs in studies may affect relative prediction level. Nonetheless, we established that (per example) the percentage variance in CPSP explained by overlap with CWP and RA, both in the discovery and replication cohorts in this study, was in the same range of that reported for genetic overlap between CWP and pelvic pain in twin studies (95% confidence interval of 3–28%) [42]. This provided sufficient confidence in the validity of our approach and outcome (suggestions to optimize setting for prospective studies have been indicated below).

### Shared mechanisms in CPSP and peripheral pain syndromes

The three CPP subtypes that showed genetic overlap with CPSP in both cohorts are known to affect peripheral nerves directly [16, 17, 43]. Sciatica involves nerve compression such as intervertebral disc rupture (the most common cause of sciatica) and other nonspinal causes of sciatica (e.g. gynaecologic causes or traumatic injury) [16]. In chronic widespread pain, both central and peripheral sensitization play a role, involving peripheral acid-sensing ion channels, decreased density of epidermal nerve fibres and proinflammatory cytokines [43, 44]. Rheumatoid arthritis (RA) originates in the immune system: pain originates from the affected joints, where inflammatory cytokines sensitize peripheral nociceptors or modify receptor activation thresholds [17]. All the processes underlying the abovementioned chronic peripheral pain syndromes have been associated with the chronification of postsurgical pain as well [45].

Chronic headache disorders (migraine and cluster headache) show a different pathophysiology. In these disorders, there is a clear involvement of the vasculature, and part of the pathophysiology seems to stem from an asynchrony in cortical processing [31, 32]. Migraine occurs mostly in women and pain develops by an interplay between vasculature, nerve innervation of both dura and skull and central nervous processing [31]. Cluster headaches (CH) classifies as a severe headache disorder occurring mostly in men, where the pathophysiology is thought to comprise synchronised abnormal activity in the hypothalamus, trigeminal vasculature and central nervous processing [32]. CGRP is a key player in both cluster headaches and migraine pathophysiology which is a potent vasodilator but was also shown to modulate activity of trigeminal neurons [31, 32]. Our genetic analyses suggest CPSP is aetiologically distinct from CH and migraine; the link between vasculature and nervous systems may explain these differences.

Consistent with the results in our study, comparative twin studies report only a low phenotypic correlation between CWP and migraine indicating them to be aetiologically distinct subgroups. However, a high correlation between CWP and low back pain was found indicative of an overlap between two different CPPs and more closely related aetiologically [42, 46]. This provides further evidence for the lack of genetic overlap between CPSP and headache-related disorders.

Comparison and genetic overlap between CPSP and osteoarthritis (OA) showed no consistent genetic overlap with CPSP between the cohorts used. This difference may point to potential involvement of additional, yet unknown genetic or environmental (e.g. sociocultural or demographic) factors between the discovery (the Netherlands) replication cohort (Italy). OA is caused by a degenerative articular cartilage condition and also involves the immune system, matrix proteins and metalloproteinases [47, 48]. The pathophysiology of the disease is diverse and complex, and frequently involves increased innervation and vascularization in the diseased joint [48]. Of note, the discovery cohort solely consisted of patients who underwent a hysterectomy, whereas the replication cohort comprised a mixture of knee and abdominal surgeries [47]. Since the knee is often affected in osteoarthritis, it is plausible that the estimated genetic overlap is affected by the surgery site, i.e. reflecting an indication for the surgery and possible postsurgical pain [47]. In this context, it is important to note that random allocation of all patients (i.e. original discovery and replication cohorts combined) over 10 fictional cohort-pairs (“discovery” vs “replication”), confirmed the outcome (i.e. PRS-based genetic overlap with CPSP) for RA and CWP, but nullified the OA findings, suggesting that the original OA findings were indeed caused by cohort-specific factors (RRIvR, data not shown). The observed inconsistency could be caused by a difference in sample size between the two target cohorts, as pointed out above. Based on these considerations, we suggest that genetic overlap found within the discovery cohort (*n* = 303) may be a more realistic estimate. For these reasons, subsequent pathway analyses were conducted without inclusion of the SNPs that predicted genetic overlap between OA and CPSP.

### Pathway analysis

GO analysis on the genetic overlap of CPSP with CPP subtypes (RA, CWP, sciatica) resulted in the identification of 4 common biological processes clusters, 3 cellular components clusters and 5 molecular functions clusters that could provide insight in shared aetiology [35, 40]. Pathway analysis of the SNPs underlying the genetic overlap between peripheral pain syndromes and CPSP indicated involvement of neuronal processes: nervous system process and neuron part sodium channel activity, and of inflammatory response: response to cytokine and response to wounding, and regulation of immune system process. These findings are consistent with published reports on the interaction of the neuronal and inflammatory reaction in the aetiology of chronic pain [49–53]. The involvement of sodium channels in pain, as conductors of action potentials and thus the nociceptive signal from the periphery towards the brain, is well-documented [54]. The most well-known example of the relation between pain and sodium channels is congenital insensitivity to pain which is caused by a mutation within the sodium channel 1.7 (Na_v_1.7) [55]. Genetic variations within sodium channels have been associated with multiple chronic pain conditions such as small fibre neuropathy, painful diabetic polyneuropathy and peripheral neuropathies [56–60]. Of note, genetic variations in genes encoding for sodium channels have not been significantly associated with CPSP before: besides the finding reported in the current study, only one earlier report investigated a sodium channel (gene *SCN9*) in relation to CPSP [61].

In CPPs, the communication between neurons and the immune system has been well documented [49–53]. This is consistent with what is known about the pathophysiology of CPPs and CPSP. Both in CPSP and CPPs, neuroinflammation (via glial cells) plays a key role in the maintenance of central sensitization [49, 62–65]. The communication between nociceptive afferents and glial cells is bidirectional, whereby both can release cytokines and chemokines that modulate the response of the other [64]. When activated, nociceptive afferents release fractalkine which binds to glial cells [62, 65]. Consequently, the glial cells release IL1β which leads to increased sodium channel activity and subsequent hyperalgesia and allodynia [62, 64, 65]. Central sensitization is a fundamental process in the chronification of pain and both neuronal signalling and inflammatory response play a key role in this process [66, 67]. Central sensitization occurs due to increased and continuous action potentials coming from the nociceptive afferents most often caused by a combination of local inflammatory processes and tissue or nerve damage [66, 67].

The genetic overlap across CPPs may ultimately be translated to clinical practice. Polygenic risk scores have been used in migraine cohorts to not only identify patients likely to develop migraine but also to identify subclusters of patients who respond to certain classes of medication [68]. This same approach was tried in psychological disorders where they combined major depression disorders and neuroticism to predict efficacy of antidepressant drugs, and although not significant, they showed that a greater genetic load for MDD and neuroticism was associated with a less favourable response to antidepressants [69]. Secondly, the PRS can be integrated into currently available clinical prediction models. In diabetes and prostate cancer, the predictive accuracy is higher than the currently available clinical models [70]. A recent clinical prediction model on CPSP increased the predictive power of by including a single SNP into the prediction model [7]. This increase in predictive power was not significant but including a complete PRS into the prediction model would significantly improve the clinical prediction modelling [52, 62, 69, 71].

### Limitations

This is the first study that combines published GWAS datasets to study genetic overlap across chronic pain phenotypes and CPSP. A limitation is the fact that the number of SNPs reported in published GWAS analyses does vary substantially between studies. Ideally, the input set of SNPs for PRS analysis is the entire GWAS dataset, as inclusion of more SNPs can lead to a better PRS score: a PRS has more predictive power if more causal SNPs are included in the combined score [33, 41]. Some studies were omitted from the current study as only the top hits were reported [72–74]. This complicates and limits accurate PRS assessment as the technique requires genome-wide input [75]. The recommendation to include all summary statistics (preferably raw data) as part of publications will enhance transparency and robustness of analyses and interpretation. A second limitation of this study is the sample size of the various studies included in the analyses. As for accurate measurements, sample sizes of above 2000 people are preferred; small sample size will lead to an inflation of the explained variance [41]. For the current analysis, two small studies (Table 1) were underpowered [9, 28]; the other studies were sufficiently powered for the analyses. To overcome the small sample size in the discovery cohort, the analysis was repeated in an independent replication cohort. The relatively high *p* value for CPSP/Sc overlap stresses the importance of increasing sample size in future research using to pinpoint the origin of genetic overlap in PRS analysis.

**Table 1:** Articles included in the PRS analysis

Author	Year	Condition	Population	Sex	Sample size (cases–controls)	SNPs reported
Van Reij et al.	2019	Chronic postsurgical pain	European Ancestry	Women	439 (45–394)	6,241,991
Gormley et al.	2016	Migraine	European ancestry	Men and women	375,752 (59,674–316,078)	7208
Bacchelli et al.	2016	Cluster headache	Italian	Men and women	458 (99–359)	14,167
Peters et al.	2013	Chronic widespread pain	European ancestry	Women	16,568 (2788–13,780)	89
Zeggini et al.	2012	Osteoarthritis	European ancestry	Men and women	50,411 (7473–42,938)	129
Plenge et al.	2007	Rheumatoid arthritis	North America and Sweden	Men and women	3372 (1522–1850)	297,081
Lemmela et al.	2016	Sciatica	Finnish	Men and women	3961 (291–3671)	380,066

The herein presented pathway analysis provides a starting point for functional studies on pathways, factors and mechanisms involved in CPSP, to substantiate the potentially shared aetiology of CPSP and CPP syndromes. An obvious candidate process involves sodium channel biochemistry. Future research aimed at understanding the impact of genetic variations on the development of CPSP should include functional aspects of genetic networks and corresponding regulatory processes in chronic pain. Functional aspects of both coding and noncoding SNPs should be elucidated to fully understand the impact of genetic variation on the development of chronic pain. Studies on the effects of genetic variation on protein function, cell signalling and cell and organismal physiology should further clarify their mechanistic connection to chronic peripheral pain syndromes, among which CPSP.

## Conclusion

In conclusion, this study is the first to report genetic overlap between regulatory processes implicated in CPSP and chronic peripheral pain syndromes (CPP). The genes identified in the genetic overlap and the factors involved in chronification of postoperative pain are related to the regulation of neurological signalling and inflammatory responses. Enhanced understanding of mechanisms underlying chronification of pain will aid the development of new preventative therapeutic strategies for CPSP.

## Electronic supplementary material

ESM 1 (DOCX 816 kb)

ESM 2 (DOCX 17 kb)

ESM 3 (XLSX 5307 kb)

ESM 4 (TXT 563 kb)

ESM 5 (TXT 57 kb)

ESM 6 (TXT 106 kb)
