# Supplementary material for: Polygenic risk scores indicates genetic overlap between peripheral pain syndromes and chronic postsurgical pain
Source: Neurogenetics. 2020 May 6;21(3):205–15. doi: 10.1007/s10048-020-00614-5 (PMC7283206; doi:10.1007/s10048-020-00614-5)
Supplement: Supplementary file 1 — (DOCX 816 kb) [file 10048_2020_614_MOESM1_ESM.docx]

Supplemental figure 1, REVIGO treemap of pathway analysis clusters of biological process gene ontologies.


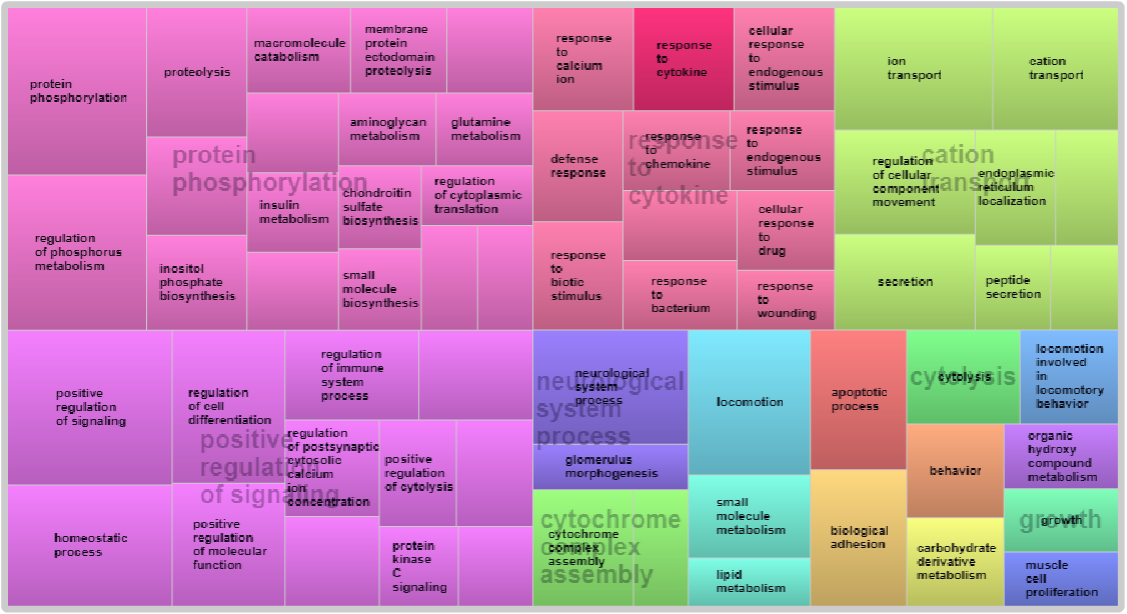


Each cluster represents similar biological process gene ontologies described by a common descriptive gene ontology term

Supplemental figure 2 REVIGO treemap of pathway analysis clusters of cellular component gene ontologies.


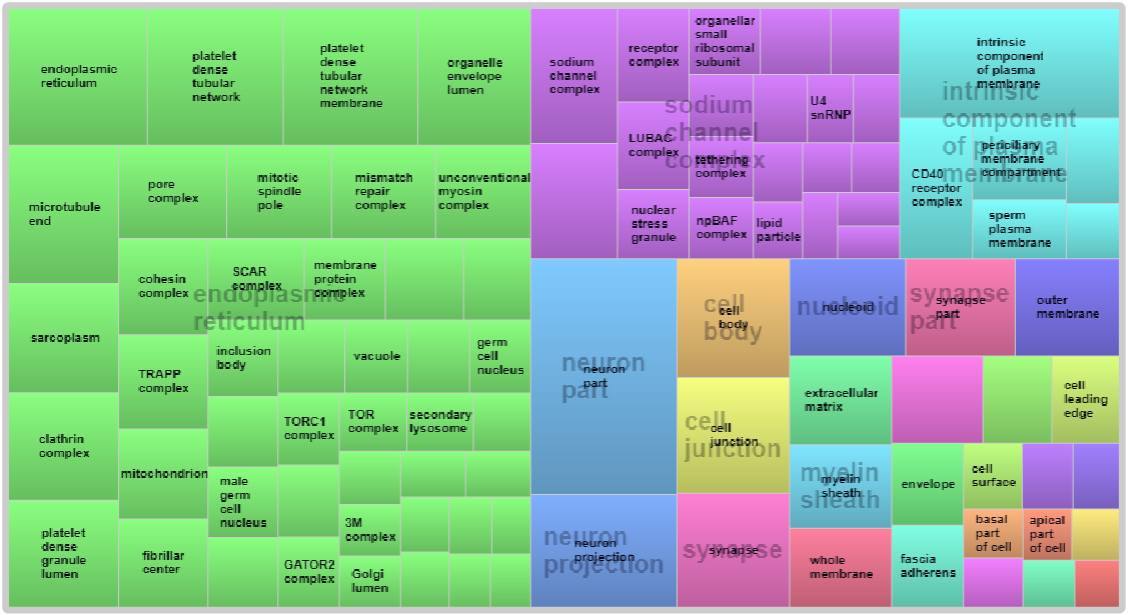


Each cluster represents similar cellular component gene ontologies described by a common descriptive gene ontology term

Supplemental figure 3 REVIGO treemap of pathway analysis clusters of Molecular Function gene ontologies.


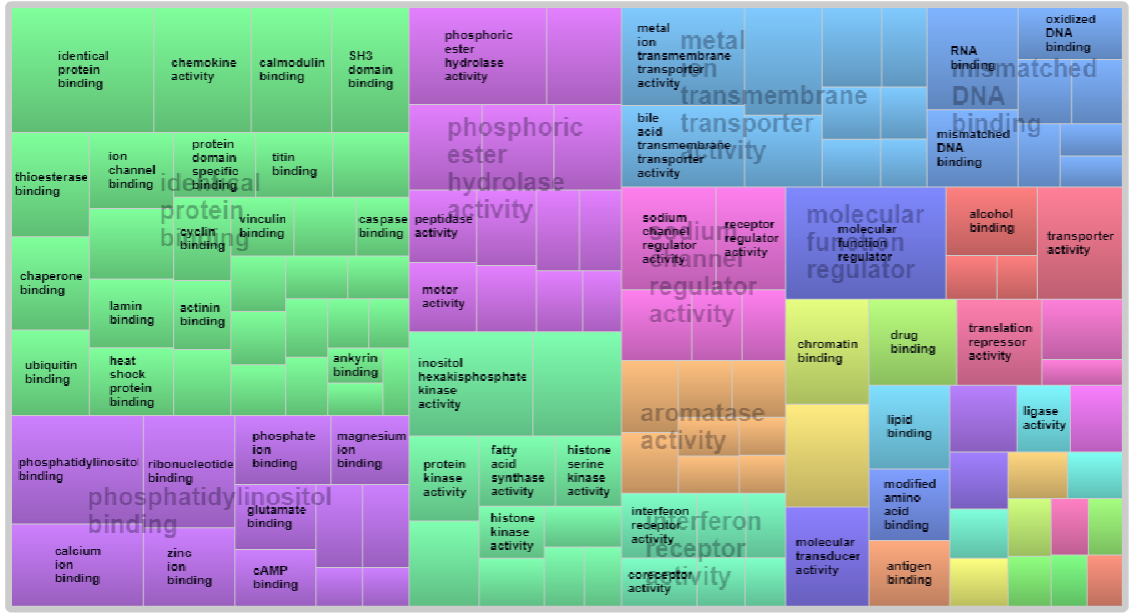


Each cluster represents similar molecular function gene ontologies described by a common descriptive gene ontology term
