# Supplementary material for: Polygenic risk scores indicates genetic overlap between peripheral pain syndromes and chronic postsurgical pain
Source: Neurogenetics. 2020 May 6;21(3):205–15. doi: 10.1007/s10048-020-00614-5 (PMC7283206; doi:10.1007/s10048-020-00614-5)
Supplement: Supplementary file 2 — (DOCX 17 kb) [file 10048_2020_614_MOESM2_ESM.docx]

Supplemental Table 1: optimal p-value results PRS scores for CPSP prediction in discovery cohort based on pain phenotypes

| Phenotype | Threshold | PRS_R^2^ | Coefficient | Standard_error | Pvalue | Num_SNP |
| --- | --- | --- | --- | --- | --- | --- |
| Cluster headache | 0.0226 | 0.014247 | 94.313 | 63.8321 | 0.14 | 3429 |
| Migraine | 0.0001 | 0.003774 | 132.164 | 174.162 | 0.448 | 214 |
| Sciatica | 0.00025 | 0.026629 | -42.692 | 21.3395 | 0.045 | 267 |
| Chronic widespread pain | 0.0875 | 0.0294922 | 0.033183 | 23.0474 | 10.8211 | 6 |
| Rheumatoid arthritis | 0.0177 | 0.03742 | 348.111 | 146.096 | 0.017 | 2771 |
| Osteoarthritis | 0.0001 | 0.007937 | -31.5548 | 28.6297 | 0.27 | 74 |

*Abbreviations:* PRS = polygenic risk score, Num_SNP = number of SNPs included in the significant polygenic risk score.

Supplemental Table 2: optimal p-value results PRS scores for CPSP prediction in replication cohort based on pain phenotypes

| Phenotype | Threshold | PRS_R^2^ | Coefficient | Standard_error | Pvalue | Num_SNP |
| --- | --- | --- | --- | --- | --- | --- |
| Cluster headache | 0.02865 | 0.020731 | 110.979 | 110.166 | 0.314 | 4557 |
| Migraine | 0.0001 | 0.016748 | 245.526 | 272.742 | 0.368 | 207 |
| Sciatica | 0.00385 | 0.0847342 | 293.388 | 146.183 | 0.0447506 | 2149 |
| Chronic widespread pain | 0.141 | 0.213118 | -95.548 | 31.8452 | 0.00269638 | 12 |
| Rheumatoid arthritis | 0.3549 | 0.232604 | -4111.97 | 1334.35 | 0.00205877 | 28117 |
| Osteoarthritis | 0.0001 | 0.113215 | -105.29 | 45.9319 | 0.022 | 76 |

*Abbreviations:* PRS = polygenic risk score, Num_SNP = number of SNPs included in the significant polygenic risk score.
